# Isolation and Characterization of CvIV4: A Pain Inducing α- Scorpion Toxin

**DOI:** 10.1371/journal.pone.0023520

**Published:** 2011-08-24

**Authors:** Ashlee H. Rowe, Yucheng Xiao, Joseph Scales, Klaus D. Linse, Matthew P. Rowe, Theodore R. Cummins, Harold H. Zakon

**Affiliations:** 1 Section of Neurobiology, University of Texas at Austin, Austin, Texas, United States of America; 2 Institute for Cell and Molecular Biology Protein and Mass Spectroscopy Facility, University of Texas at Austin, Austin, Texas, United States of America; 3 Department of Pharmacology and Toxicology, Stark Neurosciences Research Institute, Indiana University School of Medicine, Indianapolis, Indiana, United States of America; 4 Department of Biological Sciences, Sam Houston State University, Huntsville, Texas, United States of America; University of Cincinnatti, United States of America

## Abstract

**Background:**

Among scorpion species, the Buthidae produce the most deadly and painful venoms. However, little is known regarding the venom components that cause pain and their mechanism of action. Using a paw-licking assay (*Mus musculus*), this study compared the pain-inducing capabilities of venoms from two species of New World scorpion (*Centruroides vittatus*, *C. exilicauda*) belonging to the neurotoxin-producing family Buthidae with one species of non-neurotoxin producing scorpion (*Vaejovis spinigerus*) in the family Vaejovidae. A pain-inducing α-toxin (CvIV4) was isolated from the venom of *C. vittatus* and tested on five Na^+^ channel isoforms.

**Principal Findings:**

*C. vittatus* and *C. exilicauda* venoms produced significantly more paw licking in *Mus* than *V. spinigerus* venom. CvIV4 produced paw licking in *Mus* equivalent to the effects of whole venom. CvIV4 slowed the fast inactivation of Na_v_1.7, a Na^+^ channel expressed in peripheral pain-pathway neurons (nociceptors), but did not affect the Na_v_1.8-based sodium currents of these neurons. CvIV4 also slowed the fast inactivation of Na_v_1.2, Na_v_1.3 and Na_v_1.4. The effects of CvIV4 are similar to Old World α-toxins that target Na_v_1.7 (AahII, BmK MI, LqhIII, OD1), however the primary structure of CvIV4 is not similar to these toxins. Mutant Na_v_1.7 channels (D1586A and E1589Q, DIV S3–S4 linker) reduced but did not abolish the effects of CvIV4.

**Conclusions:**

This study: 1) agrees with anecdotal evidence suggesting that buthid venom is significantly more painful than non-neurotoxic venom; 2) demonstrates that New World buthids inflict painful stings via toxins that modulate Na^+^ channels expressed in nociceptors; 3) reveals that Old and New World buthids employ similar mechanisms to produce pain. Old and New World α-toxins that target Na_v_1.7 have diverged in sequence, but the activity of these toxins is similar. Pain-inducing toxins may have evolved in a common ancestor. Alternatively, these toxins may be the product of convergent evolution.

## Introduction

For animals that lack the advantage of size, razor-like claws, speed, camouflage, etc. to overpower or outmaneuver their predators, painful venom can serve as a potent weapon. A diversity of animals including ants, wasps, bees, scorpions, spiders, snakes, jellyfish, stonefish, and stingrays employ painful venom to either deter their enemies or escape subjugation.

Among all species of scorpion, those in the family Buthidae produce the world's most deadly venoms [1]. Buthid venom is a mixture of several peptides that bind different families of ion channels (Na^+^, K^+^, Cl^−^, Ca^2+^) in excitable membranes of nerve and muscle [2], [3], [4], [5]. The majority of toxins that have been described recognize either sodium (Na^+^) or potassium (K^+^) channels. Toxins that bind Na^+^ channels alter the gating mechanism, making the channel likely to open near the resting membrane potential and then inhibiting fast inactivation, thus prolonging the flow of Na^+^ ions through the pore [6], [7]. Toxins that bind K^+^ channels block the flow of K^+^ ions through the channel, preventing the membrane from returning to its resting potential after depolarization [8], [9]. The synergistic effect of these toxins is hyper-excitability of nerve and muscle cells that can cause a wide range of physiological malfunction [10], [11], [12], [13]. Even when buthid stings are not fatal, humans report excruciating pain that may last from several hours to days. While the buthid toxins that cause seizures, paralysis and respiratory failure have been well studied, little is known regarding the venom components that cause pain and their mechanism of action.

Animals sense pain when peripheral sensory neurons (nociceptors) are activated [14], [15] and transmit information about noxious stimuli to the central nervous system (CNS). The cell bodies of nociceptors are housed in dorsal root ganglia (DRG), located just outside the spinal cord. A number of distinct DRG-expressed voltage-gated sodium channels (VGSCs), primarily Na_v_1.7, Na_v_1.8, Na_v_1.9, play a major role in transducing noxious stimuli in animals.

Given that buthid scorpions produce toxins that bind Na^+^ channels in excitable membranes, it is plausible that their venom induces pain by initiating action potentials in nociceptors. Because some human pain disorders involve Na^+^ channels expressed in nociceptors [14], [15], there has been an effort, albeit limited, to determine the components in buthid venom that induce pain with the goal of isolating peptides that discriminate among the DRG-expressed VGSCs. For example, BmK I, isolated from the venom of *Buthus martensii* Karsch, an Old World buthid (species that originated in Africa and Asia), induces paw licking when injected into the hind paws of rats. BmK I modulates DRG-expressed Na^+^ currents in rat, but the specific ion-channel target was not identified [16], [17], [18]. A separate study showed that BmK MI (synonym for BmK I) slows the fast inactivation of Na_v_1.7 expressed in *Xenopus* ooctyes [19]. Toxins isolated from the Old World buthids *Odonthobuthus doriae* (ODI), *Androctonus australis* Hector (AahII) and *Leiurus quinquestriatus hebraeus* (LqhIII) also slow the fast inactivation of Na_v_1.7 [19], [20]. However, while the venoms of these three scorpions are reported to be painful, ODI, AahII and LqhIII were not tested for their ability to induce paw licking in a rodent model. Collectively, the results from these studies support the hypothesis that Old World buthids produce painful stings, in part, by toxins that modulate DRG-expressed Na^+^ channels. New World buthids (species that originated in North and South America) are reported to produce intensely painful stings, however, to our knowledge no studies have identified pain-inducing components from the venoms of New World buthids.

The goal of this study was to gain a better understanding of how neurotoxic venom produced by buthid scorpions induces pain in mammals. Our objectives were to 1) establish in a mouse model whether venom produced by buthid scorpions is more painful than venom produced by scorpions from other families as anecdotally reported; 2) determine whether Na^+^ channel toxins are involved in generating the intense pain produced by New World buthids, and if so, identify their ion-channel targets and mechanism of action; and 3) compare pain-inducing toxins from Old and New World buthids to determine whether they employ similar venom components to produce painful stings.

To achieve these objectives, we measured the duration of paw licking by *Mus musculus* in response to injections of venom or venom fractions into their hind paws. While we do not know what mice perceive, we assume that mice lick their paws in response to pain. Thus, we will refer to the venom and toxins that produce paw licking as “painful” or “pain inducing.”

## Materials and Methods

This study was carried out in strict accordance with recommendations in the Guide for the Care and Use of Laboratory Animals of the National Institutes of Health. Protocols were approved by the Institutional Animal Care and Use Committees at the University of Texas at Austin, protocol number AUP-2009-00027, and the Indiana University School of Medicine, protocol number 3552. All efforts were made to reduce the number of animals used and to minimize the suffering of animals.

### Scorpion collection

Specimens of *C. exilicauda* (synonym for *C. sculpturatus*) and *V. spinigerus* were collected from the foothills of the Santa Rita Mountains, AZ. Specimens of *C. vittatus* were collected from the foothills of the Organ Mountains, NM. Scorpions were collected at night using ultraviolet light and then placed in plastic bags for transport. Scorpions were housed in plastic containers (52 cm L×35 cm W×15.6 cm H) in a room with a 12/12 light cycle and daily temperature of 25–26°C. Aquarium gravel was used to line the containers and cardboard egg crates were added to provide the scorpions with a refuge. Scorpions were fed live crickets once a week and provided with water *ad libitum*.

### Venom extraction and preparation

Fresh venom was extracted from captive scorpions using electrical stimulation of the telson (terminal tail segment that houses the venom gland). The crude venom was dissolved in sterile water and centrifuged at 14,500 RPM, 4°C, for 15 minutes to remove insoluble components. The supernatant was collected and the protein concentration determined using a nanodrop spectrophotometer. For protocols isolating pain-inducing peptides from venom, aliquots of the supernatant were stored for a short period of time at −80° C and then thawed before applying the sample to the perfusion column. For behavioral assays to screen venom and venom fractions for pain-inducing capability, aliquots of soluble venom were lyophilized and maintained at −20° C until tested.

### Isolation of peptides that induce paw licking in mice

Venom peptides were purified by tandem purification. Soluble, whole venom from *C. vittatus* was separated into fractions using perfusion chromatography (1 dimension). Aliquots of whole venom were injected into a POROS (PerSeptive Biosystems, Framingham, MA) R2 10 µm perfusion column (4.6 mm internal diameter×100 mm length). The perfusion column was connected to a Bio Rad (Hercules, CA) Biologic Duoflow Maximizer system with a Quad Tec UV-Vis detector. Fractions were separated using the following method: linear gradient with 0% to 4% solvent B at 0.80 ml/min for 2.0 ml; linear gradient with 8% to 38% solvent B at 0.80 ml/min for 80.0 ml; linear gradient with 44% to 100% solvent B at 0.80 ml/min for 10.0 ml; isocratic flow with 0% solvent A, 100% solvent B at 0.80 ml/min for 3.0 ml; linear gradient with 100% to 0% solvent B at 0.80 ml/min for 3.0 ml; isocratic flow with 100% solvent A, 0% solvent B at 0.80 ml/min for 4.0 ml (solvent A, 0.1% trifluoroacetic acid in LC water; solvent B, acetonitrile). The elution of each fraction was monitored by following the UV trace at 214 nm and 280 nm. Fractions were collected manually and screened for their ability to produce pain using a behavioral assay (see below). Fractions that produced pain were further separated into individual peptides using a TARGA (Higgins Analytical, Inc., Mountain View, CA) reverse phase C_18_ column (4.6 mm internal diameter×250 mm length) (2 dimension) and a different buffer system. Individual peptides were separated using the following method: linear gradient with 0% to 8% solvent B at 0.80 ml/min for 2.0 ml; linear gradient with 8% to 44% solvent B at 0.80 ml/min for 80.0 ml; linear gradient with 44% to 100% solvent B at 0.80 ml/min for 10.0 ml; isocratic flow with 0% solvent A, 100% solvent B at 0.80 ml/min for 3.0 ml; linear gradient with 100% to 0% solvent B at 0.80 ml/min for 3.0 ml; isocratic flow with 100% solvent A, 0% solvent B at 0.80 ml/min for 4.0 ml (solvent A, 100 mM ammonium acetate, pH 6.5, in LC water; solvent B, acetonitrile). Peptides were collected manually by following the UV trace at 214 nm and 280 nm. The purity of peptides was confirmed using analytical HPLC (data not shown). Purified peptides were tested for their ability to induce pain using a behavioral assay. All peptides were further characterized using mass spectrometry.

### Behavioral assays

Soluble venom, venom fractions and purified peptides were tested for their ability to produce pain using the paw-licking assay [16], [21]. For all behavioral assays, lyophilized samples of venom or venom components were hydrated in sterile water to the final concentration and injected subcutaneously into the plantar region of the left hind paw of house mice (*Mus musculus domesticus*). To control for the pain of an injection, aliquots of an equal volume of sterile water were injected into the hind paw of an additional group of mice. Immediately following the injection, mice were placed in a Plexiglas container (8 cm W×38 cm L×26 cm H) and their response was videotaped using a digital video camcorder (Canon XL1 mini DV) equipped with a 3× wide-angle zoom lens (Canon XL 3.4–10.2 mm). The amount of time mice spent licking their paws was measured and used as an index of pain.

In the first paw-licking assay comparing the pain-inducing capability of soluble venom from three different species of scorpion, samples of lyophilized venom were diluted to a concentration of 1.7 µg/µl. An aliquot of 10 µl was injected into the hind paw of male mice (strain CD-1, 37–40 g, n = 8 per treatment) and paw licking was recorded for 10 minutes. Data are reported as the mean values in seconds (s) ±1 standard error of the mean (SE). A single factor analysis of variance (ANOVA, JMP®8, www.jmp.com) was used to test for significant effects across treatment groups. Planned orthogonal contrasts were used to test for differences between treatments.

In the behavioral assays screening *C. vittatus* venom fractions and purified peptides for pain-inducing ability, lyophilized venom fractions or peptides were diluted to a concentration of 2.0 µg/µl. A 10 µl sample was injected into the hind paw of female mice (strain CD-1, 20–24 g, n = 2–5 mice per treatment) and paw licking was recorded for 5 minutes. Paw licking values for *C. vittatus* venom fractions are reported as the mean (s) ±1 SE. A single factor ANOVA (JMP®8, www.jmp.com) was used to detect significant differences in paw licking across all treatment groups. A multiple comparisons test (Tukey's HSD) was used to identify venom fractions that produced as much paw licking as the sample of whole venom. In order to reduce the number of mice used to test samples and to conserve the limited supply of purified peptides, only two mice were used to test each individual peptide isolated from venom fractions. Measures of pain for purified peptides are shown as paw licking values (s) from each of two tests for each peptide.

### Mass spectrometry analysis

The molecular mass of *C. vittatus* venom fractions and purified peptides were estimated using matrix assisted laser desorption ionization time of flight (MALDI TOF) technology. Mass spectrometry was performed using an Applied Biosystems (Fullerton, CA) Voyager Biospectrometry Workstation MALDI-TOF mass spectrometer in the Institute of Cellular and Molecular Biology Protein Microanalysis Facility of the University of Texas at Austin. Aliquots of peptide samples in aqueous solution or containing up to 50% acetonitrile were combined with freshly prepared matrix solution [saturated sinapinic acid dissolved in a mixture of 50 or 75% (v/v) acetonitrile, 0.3% (v/v) trifluoroacetic acid (TFA) and distilled water]. Mass spectral measurements were made with ratios of peptide solution to matrix solution ranging from 1∶1 to 1∶6. The optimum ratio was typically 1∶4, thus, all reported spectra were made with a ratio of 1∶4. Sample aliquots of 0.5 or 1.0 mL were spotted onto stainless steel sample plates and spectra were collected by averaging 10–20 laser shots. Samples were irradiated with a nitrogen laser (Laser Science Inc.) operated at 337 nm, attenuated and focused on the sample target using the built-in Perseptive GRAMS/386 software. Ions were accelerated with a deflection voltage of 30 kV and differentiated according to their m/z using a time-of-flight mass analyzer. Myoglobin (horse heart; Mr 16,950.7), insulin (Mr 5,733.5) and Bradykinin (Mr 1,060.2) were used as external standards to calibrate the spectra.

### Amino acid analysis (N-terminal Protein Sequencing)

After confirming the purity of the peptides isolated from *C. vittatus*' venom, the N-terminal amino acid sequence for each peptide was determined using Edman degradation. Automated protein sequencing was performed on a 492A protein sequencer equipped with a 120A HPLC system (PE Applied Biosystems). All reagents and solvents used for the sequencer were obtained from PE Applied Biosystems. Aliquots of each peptide were reduced with DTT and chemically modified with iodoacetamide. Briefly, peptides were incubated with 100 mM DTT at 37°C for one hour followed by incubation with 120 mM iodoacetamide at 37°C for one hour. The peptides were then spotted onto either polybrene (Bioprene) treated glass fiber disks or PVDF membrane pieces (ca. 1×1 mm). PVDF membrane pieces were wetted with neat methanol and an aliquot of a 1 to 20 dilution of the Bioprene solution while glass fiber disks were treated only with the polybrene solution. Both the glass fiber disks and membrane pieces were dried under a stream of nitrogen and then loaded into the reaction cartridge for sequencing.

### Molecular Analyses: cloning CvIV4 venom gland cDNA

#### RNA extraction and cDNA synthesis

Two female specimens of *C. vittatus* (Organ Mountains, NM) provided the RNA for molecular analyses of the gene that encodes toxin CvIV4. Total RNA was extracted from the venom gland of scorpions 24 hours following venom extraction. The telson was removed from each scorpion and immediately frozen at −80° C. Frozen telsons were homogenized in RNA STAT-60 (Tel-Test, Inc., Friendswood, TX). Total RNA was isolated from the homogenate according to the manufacturer's guidelines. Complementary DNA (cDNA) was generated from approximately 500 ng of total RNA. Oligo d(T)_20_ (www.invitrogen.com) was used to prime the polyadenylated (poly A+) mRNA and Invitrogen SuperScript® III Reverse Transcriptase (www.invitrogen.com) was used to reverse transcribe the mRNA.

#### Amplification of cDNA encoding CvIV4

The cDNA prepared from *C. vittatus* venom gland RNA served as the template to amplify the gene that encodes CvIV4. The polymerase chain reaction (PCR) procedure was used to amplify cDNA in three steps. In the first step, degenerate primers (1 and 2, Table 1) were used to amplify the initial 148 nucleotides from the gene that correspond to the beginning of the mature toxin. Degenerate primers were designed from a combination of CvIV4 peptide sequence (direct sequencing of peptide, Edman degradation) and published scorpion toxin nucleotide sequences (NCBI). In the second step, nested gene-specific forward primers (3 and 4, Table 1), designed from the PCR product obtained during the first step, were paired with a custom designed oligo d(T)_24_VN reverse primer (7, Table 1, Integrated DNA Technologies, www.idtdna.com) to amplify nucleotide sequence corresponding to the mature toxin and the three prime untranslated region (3′ UTR). In the third step, nested gene-specific reverse primers (5 and 6, Table 1), designed from the PCR products obtained during the first and second steps, were paired with 5′ RACE (rapid amplification of cDNA ends) nested forward primers (see manufacturer's protocol for inner and outer primer sequence, Ambion FirstChoice® RLM-RACE, www.appliedbiosystems.com) to amplify nucleotide sequence corresponding to the mature toxin, the signal peptide and the 5′ UTR. All PCR procedures used TaKaRa Ex Taq™ polymerase (www.takara-bio.com) to amplify the cDNA template according to the manufacturer's directions. PCR amplifications of gene sequence were conducted using an Eppendorf Thermocyler and the products were analyzed on 1% agarose gels. DNA fragments were extracted from the agarose gels and purified using Invitrogen PureLink™ Quick Gel Extraction Kit (www.invitrogen.com).

**Table 1:** Primers for Amplification of cDNA Encoding CvIV4.

No.	Sequence	Direction	1Position
1	AARAARGAYGGNTAYCCNGTNGAN	Forward	100–123
2	CVYTATCSGGWAGVCCKWVRCART	Reverse	240–217
3	CACAGTGGTTGCAAATATACTTGTTGGAAA	Forward	124–153
4	ACTTGTTGGAAAAACGAATATTGT	Forward	141–165
5	TTTGTTTTTAAAGGTACGTTATCAGGAAGACCTGT	Reverse	266–232
6	TGTTTACTTCCTTTTACCGTTACA	Reverse	297–274
7	TTTTTTTTTTTTTTTTTTTTTTTVN	Reverse	389–362

1 Position of primer oligonucleotides with respect to CvIV4 cDNA sequence shown in Fig. 4B.

#### Cloning and sequencing PCR products

*Taq* polymerase amplified products from steps 1–3 were inserted into a plasmid vector (pCR®4-TOPO, TOPO TA Cloning® Kit for Sequencing, www.invitrogen.com). Plasmid vectors with DNA inserts were used to transform *E. coli* cells (One Shot® TOP10 Competent Cells, www.invitrogen.com). Cloning and transformation procedures were conducted following the manufacturer's guidelines. Colonies were selected and grown overnight in LB medium containing 50 µg/ ml kanamycin. *E. coli* cells were harvested from overnight cultures and plasmids with DNA inserts were extracted and purified using Qiagen's QIAprep Spin Miniprep Kit (www.qiagen.com). DNA samples were eluted in nuclease-free water and sequenced in both directions using M13 Forward and M13 Reverse primers supplied with the TOPO TA cloning kit. DNA samples were sequenced at the University of Texas at Austin Institute for Cell & Molecular Biology (ICMB) Sequencing Facility using capillary-based Applied Biosystems 3730 and 3130 automated DNA Analyzers. The nucleotide (cDNA) sequence encoding a clone of CvIV4 has been deposited to the NCBI GenBank (accession number JF938594).

### Electrophysiological recordings

#### Plasmids of sodium channels

The cDNA genes encoding rat (r) Na_v_1.2, rNa_v_1.3 and rNa_v_1.4 were inserted into the vectors pRC-CMV, pcDNA3.1-mod and pRBG4, respectively [22], [23], [24]. The cDNA genes encoding human (h) Na_v_1.5 and hNa_v_1.7 were subcloned into the vectors pcDNA3.1 and pcDNA3.1-mod, respectively [25].

#### Preparation of Stably Transfected Cell Lines

HEK293 cells were obtained from ATCC, Manassas, VA, USA. Use of the HEK293 cells was approved by the Institutional Biosafety Committee and conformed to the ethical guidelines for the National Institutes of Health for the use of human-derived cell lines. The transfections of all wild type sodium channels (Na_v_1.2, Na_v_1.3, Na_v_1.4, Na_v_1.5, and Na_v_1.7) were carried out using the calcium phosphate precipitation method as described by Xiao et al. (2010). However, no β subunit or green fluorescent protein reporter plasmid was included in the calcium phosphate-DNA mixture. After transfection of Human Embryonic Kidney cells (HEK) for 15–20 h, the cells were washed with fresh medium. After 48 h, antibiotic (G418, Geneticin; Cellgro, Herndon, VA) was added to select for neomycin-resistant cells. After 2–3 weeks in G418, colonies were picked, split, and subsequently tested for channel expression using whole-cell patch clamp recording techniques.

#### Dorsal root ganglion (DRG) neuron preparation

Adult rat DRG neurons were acutely dissociated and cultured as previously described [26]. Briefly, rats were anesthetized by exposure to CO_2_ and decapitated. Cells were treated with collagenase (1 mg/ml) and papain (1 mg/ml), dissociated in DMEM supplemented with 10% fetal bovine serum, and plated on glass coverslips coated with polyornithine and laminin. Cultures were maintained at 37°C in a 5% CO_2_ incubator, and media was changed every 2 days during experimental incubation periods. DRG neurons express both tetrodotoxin-sensitive (TTX-S) and TTX-resistant (TTX-R) sodium channels. In order to isolate TTX-R sodium current, DRG neurons were pretreated with 500 nM TTX to block TTX-S sodium current.

#### Site-directed mutagenesis

Two hNa_v_1.7 mutations (D1586A and E1589Q, DIV S3–S4 loop) were constructed using the QuikChange II XL Site-Directed Mutagenesis kit (Stratagene, La Jolla, CA) and expressed in HEK293 cells as described by Xiao et al. (2010).

#### Whole-cell Patch Clamp Recordings

Whole-cell patch clamp recordings were performed at room temperature (∼21°C) using an EPC-10 amplifier (HEKA, Lambrecht, Germany). Data were acquired on a Pentium IV computer using the Pulse program (version 8.31; HEKA). Fire-polished electrodes were fabricated from 1.7-mm capillary glass (VWR, West Chester, PA) using a P-97 puller (Sutter, Novato, CA). The standard pipette solution contained (in mM): 140 CsF, 1 EGTA, 10 NaCl and 10 HEPES, pH 7.3. The standard bathing solution was (in mM): 140 NaCl, 3 KCl, 1 MgCl2, 1 CaCl2 and 10 HEPES, pH 7.3. After filling with pipette solution, the access resistance of electrode pipette ranged from 0.8 to 1.4 MΩ. The liquid junction potential for these solutions was <8 mV; data were not corrected to account for this offset. The offset potential was zeroed before contacting the cell. After establishing the whole-cell recording configuration, the resting potential was held at −100 mV for 5 min to allow adequate equilibration between the micropipette solution and the cell interior. Linear leak subtraction, based on resistance estimates from four to five hyperpolarizing pulses applied before the depolarizing test potential, was used for all voltage clamp recordings. Membrane currents were usually filtered at 5 kHz and sampled at 20 kHz. Voltage errors were minimized using 80% series resistance compensation, and the capacitance artifact was canceled using the computer-controlled circuitry of the patch clamp amplifier. The CvIV4 stock solution was made at 0.1 mM using bathing solution containing 1 mg/ml BSA, and aliquots were stored at −20°C. Before use, the solution was diluted to the desired concentration with fresh bathing solution. Toxin (30 µl) was added directly to the recording chamber (volume of 300 µl) and mixed by repeatedly pipetting to achieve the specified final concentration.

#### Data Analysis

Data were analyzed using the Pulsefit (HEKA) and GraphPad Prism 4 (GraphPad Software) programs. All data points are shown as the mean ± SE and *n* represents the number of separate experimental cells. Steady-state activation and inactivation curves were fitted using Boltzmann equation: *y* = 1/(1+exp((*V_1/2_*−*V*)/*k*), in which *V_1/2_*, *V* and *k* represented midpoint voltage of kinetics, test potential and slope factor, respectively. Dose-response curves to determine *EC_50_* values were fitted using the Hill equation: *y* = *f_TOP_*(1+10∧((log*EC_50_*−[Tx]/*EC_50_*)*^nH^*), where *nH* is Hill coefficient, *EC_50_* is half maximal effective concentration, and *f*_TOP_ is the fraction of current sensitive to inhibition at high toxin (Tx) concentration.

## Results

### Effects of scorpion venom on paw-licking behavior in *Mus*

**Figure 1:**
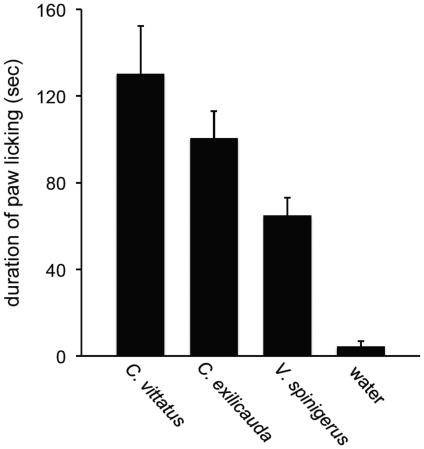
Mean (+1SE) duration of paw licking for *Mus musculus* injected with scorpion venom or water. Samples of whole, soluble venom from three scorpion species induced more hind-paw licking in *M. musculus* than the water control during a ten-minute test period following the injection (F = 37.25; df = 1, 28; P<0.0001). However, *Centruroides'* venom induced more paw licking than *V. spinigerus* venom (F = 9.49; df = 1, 28; P = 0.0046). There was no statistical difference in the duration of paw licking induced by *C. vittatus* and *C. exilicauda* venom (F = 2.47; df = 1, 28; P = 0.1275).

Tests quantifying the effects of the venoms of three different species of scorpions on paw-licking behavior in *M. musculus* (strain CD-1) showed that scorpion venom induces pain-related behaviors in mice (Fig. 1). The amount of time mice spent licking their paws in response to an injection of scorpion venom or water differed among treatment groups (for all treatment groups n = number of mice; *C. vittatus*, 130.23 sec±22.08, n = 8; *C. exilicauda*, 100.53 sec±12.42, n = 8; *V. spinigerus*, 64.94 sec±8.14, n = 8; water, 4.36 sec±2.57, n = 8; Fig. 1). A single factor ANOVA showed a significant effect across treatment groups (F = 16.40; df = 3, 28; P<0.0001). Planned orthogonal contrasts demonstrated that while the venom of all three species of scorpion produced significantly more paw licking in mice than the water control (F = 37.25; df = 1, 28; P<0.0001), the venom of both species of *Centruroides* produced significantly more paw licking than *V. spinigerus* venom (F = 9.49; df = 1, 28; P = 0.0046). Although the venom of *C. vittatus* induced more paw licking than that of *C. exilicauda*, the difference was not significant (F = 2.47; df = 1, 28; P = 0.1275). These results indicate that scorpion venom, at least the venom of the three species we tested, is painful, not only to humans, but also rodents. Moreover, the results show differences in the pain-inducing capability of different species of scorpion, and they confirm anecdotal reports that the venom of *Centruroides* is especially painful, whereas that of *V. spinigerus* is much less so.

### Isolation and identification of pain-inducing peptides

**Figure 2:**
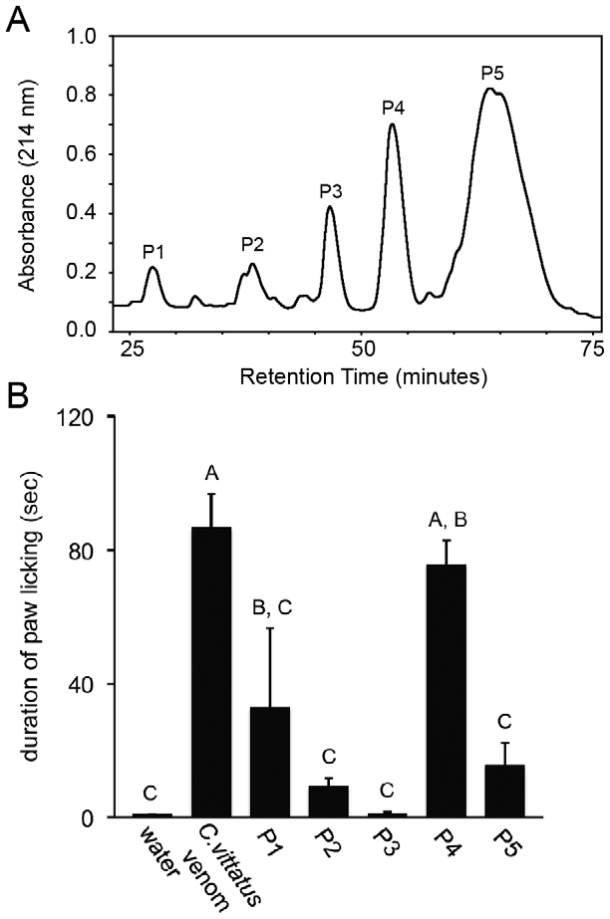
Effect of *C. vittatus* venom and venom fractions on paw-licking behavior in *Mus musculus*. A. High performance liquid chromatography (HPLC) profile of *C. vittatus* venom fractions. Whole, soluble venom from *C. vittatus* was separated into five fractions (peaks: P1, P2, P3, P4, P5). Each fraction was isolated and tested for pain using the paw-licking assay in *M. musculus*. B. Mean (+1SE) duration of paw licking for *M. musculus* injected with water, scorpion venom, or venom fractions. Paw licking was recorded for five minutes following the injection. P4 was the only fraction that was significantly more painful than water (P<0.0001) and P4 induced as much pain as whole venom (P = 0.9525). Histograms showing the same letter did not differ at the P<0.05 level of significance using Tukey's HSD test.

Because the venom of *C. vittatus* produced more paw licking than that of *C. exilicauda*, we selected *C. vittatus* venom for subsequent studies aimed at identifying the components in scorpion venom that cause pain. High performance liquid chromatography (HPLC) was used to separate *C. vittatus* whole, soluble venom into five fractions, P1–P5 (Fig. 2A). Samples of each fraction were tested for pain using the paw-licking assay on *M. musculus*. The amount of time mice spent licking their paws in response to an injection of water, scorpion venom, or venom fractions differed significantly among treatment groups (for all treatment groups n = number of mice; water, 0.11 sec±8.35, n = 5; *C. vittatus* venom, 86.87 sec±7.62, n = 6; P1, 33.00 sec±10.77, n = 3; P2, 9.33 sec±10.77, n = 3; P3, 1.23 sec±7.05, n = 7; P4, 75.73 sec±8.35, n = 5; P5, 15.66 sec±10.77, n = 3; Fig. 2B) as confirmed by an ANOVA test (F = 19.89; df = 6, 25; P<0.0001). Multiple comparisons using Tukey's HSD identified a single venom fraction, P4, that produced as much pain as whole venom (P = 0.9525; histograms representing fractions that did not differ at the P<0.05 level of significance using Tukey's HSD test were labeled with the same upper case letter; Fig. 2B). Fraction P4 was also the only fraction that was significantly more painful than water (P<0.0001).

**Figure 3:**
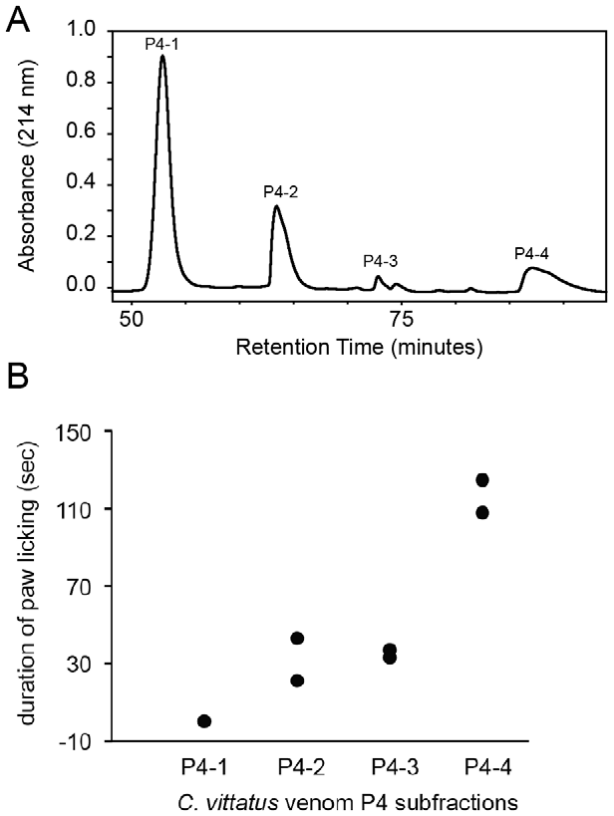
Effect of *C. vittatus* venom P4 subfractions on paw-licking behavior in *Mus musculus*. A. High performance liquid chromatography (HPLC) profile of *C. vittatus* venom P4 subfractions. Fraction P4 was separated into four subfractions (P4-1, P4-2, P4-3, P4-4). B. Duration of hind-paw licking by *M. musculus* injected with *C. vittatus* P4 subfractions. Each sample was tested on two mice and each mouse was injected only once. Paw licking was recorded for five minutes following the injection. The paw licking values from both the first and second tests are shown in plot. Note, values for the first and second tests for P4-1 are identical and markers overlap.

Mass spectrometry analysis showed that P4 contained three to four different components (data not shown). HPLC separation of P4 produced four subfractions, P4-1–P4-4 (Fig. 3A). Samples of each subfraction were isolated and tested for pain in *M. musculus* using the paw licking assay. To reduce the amount of sample consumed and the number of mice used, only two tests were conducted per subfraction. Each sample was tested on two mice and each mouse was injected only once. The four subfractions differed dramatically in the duration of paw licking they produced. Results from the first and second tests showed that subfraction P4-1 had no effect on paw licking, P4-2 and P4-3 induced a brief amount of paw licking, and P4-4 produced the longest duration of paw licking (Fig. 3B). There also appear to be differences in the latency to induce paw licking in the three subfractions causing pain. For example, P4-2 produced paw licking in each of two test mice 15 seconds after injection of the sample. P4-3 produced paw licking 10 seconds and 30 seconds after injecting the sample into the first and second test mice, respectively. P4-4 produced paw licking at 60 seconds after injection into the first test mouse and 30 seconds after injection into the second test mouse. Paw licking increased during the second minute of the test and peaked from three to five minutes. Mice injected with P4-4 were still licking their paws at the end of the five-minute test period.

### Mass spectrometry analysis

We used matrix-assisted laser desorption ionization time of flight (MALDI TOF) technology to determine the molecular mass of P4-4. Mass spectrometry analysis showed that subfraction P4-4 is a single peptide with a molecular mass of 6904.2 atomic mass units (amu, data not shown). These results demonstrate that P4-4 has a mass characteristic of scorpion toxins that bind Na^+^ channels. Scorpion Na^+^ channel toxins, also known as long-chain toxins, are polypeptides that have a mass ranging from 6500 to 8500 amu [6]. This suggests that peptides in the venom of *C. vittatus* that produce pain may function by binding Na^+^ channels expressed in pain-sensing neurons.

### Amino acid sequence determination

**Figure 4:**
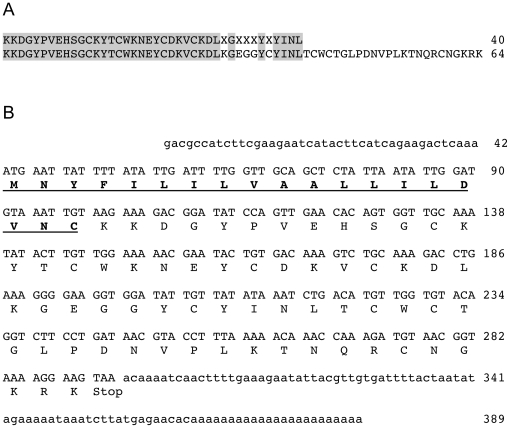
Amino acid sequence of subfraction P4-4 (CvIV4) and *C. vittatus* venom gland cDNA that encodes toxin CvIV4. A. Comparison of amino acid sequences representing CvIV4. The upper sequence represents the initial 40 amino acids of the purified peptide obtained from Edman degradation. Amino acid residues that could not be determined are shown as “X”. The lower sequence represents the translation of nucleotides from the cDNA encoding CvIV4 isolated from *C. vittatus* venom gland. Sequences representing the peptide and translated gene are identical with the exception of the five amino acid residues (X's) that could not be identified. B. Nucleotide sequence from venom gland cDNA that encodes toxin CvIV4. Nucleotide sequence from the 5′ and 3′ untranslated region (UTR) is shown as lower case letters. Sequence from the mature peptide is shown as upper case letters. Amino acid residues translated from nucleotide sequence are shown as upper case letters positioned below their corresponding codons. Amino acids designating the signal peptide are underlined and shown in bold.

Edman degradation provided the initial 40 amino acids from the N-terminal sequence of P4-4 (Fig. 4A). A BLAST (NCBI) search using the N-terminal sequence confirmed that P4-4 has a primary structure that is characteristic of scorpion toxins that bind Na^+^ channels. *C. vittatus* P4-4 was then assigned the name CvIV4 following the general guidelines for nomenclature of scorpion Na^+^ channel toxins.

### Isolation of venom gland mRNA encoding CvIV4

A comparison of the N-terminal sequence for CvIV4 with other scorpion Na^+^ channel toxins revealed that CvIV4 has a primary structure similar to alpha (α) toxins. The first seven amino acid residues and the position of the first four cysteines are identical to a number of scorpion toxins classified as α-toxins. However, CvIV4 also contains sequence that is unique. Given that the number of amino acid residues identified from direct sequencing of a peptide is limited, we isolated mRNA from the venom gland and sequenced the cDNA that encodes CvIV4 so that we could compare its structure with other scorpion α-toxins. Total RNA was isolated from the venom gland of two specimens of *C. vittatus* and reverse transcribed to produce cDNA. The gene encoding CvIV4 (Fig. 4B) was cloned and sequenced from this cDNA sample in three steps. In the first step, degenerate primers (see Table 1 for all primer sequences) designed from scorpion Na^+^ channel toxin sequences (NCBI) were used to amplify gene sequence corresponding to the initial 148 nucleotides of the mature toxin (approximately position 99-249, Fig. 4B). In the second step, nested gene-specific forward primers were designed from the nucleotide sequence obtained during the first step. These forward primers were paired with an anchored oligo d(T) reverse primer to amplify the gene from the middle of the toxin to the poly-A tail (approximately position 124 to 389, Fig. 4B). In the third step, nested gene-specific reverse primers were designed from nucleotide sequence obtained during the first and second steps. Nested gene-specific reverse primers were paired with 5′ RACE forward primers to amplify the gene from the middle of the toxin to the 5′ UTR (approximately position 1 to 297, Fig. 4B).

**Figure 5:**
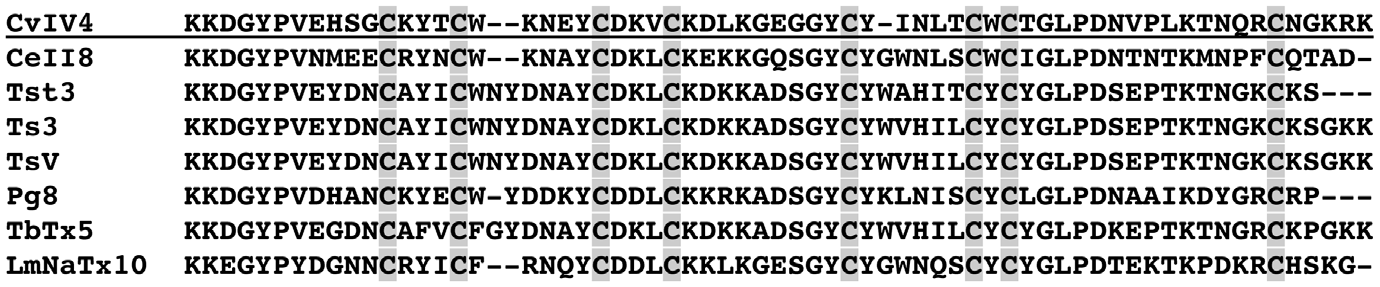
Comparison of CvIV4 translated cDNA with Na^+^ channel toxin sequence from other scorpion species. CvIV4 (underlined) is aligned with seven toxin sequences from other species. Alignment is based on cysteine residue position (shaded background) and toxins are arranged in order of descending percent identity with respect to CvIV4. Percent identify (%ID) is estimated from the number of amino acids shared by two toxins (NCBI Protein BLAST). While CvIV4 (JF938594) and CeII8 (P0CH40) are structurally similar (64% ID), they are functionally different as CvIV4 is classified as an α-toxin and CeII8 as a β-toxin (classification based on electrophysiological recordings from Na^+^ channel subtypes). CvIV4 shares over 50% of its amino acid residues with the remaining six toxins. Ts3 (P01496) and TsV (P46115) are classified as α-toxins based on electrophysiological studies of mammalian cells and tissues. Tst3 (P0C8X5), Pg8 (ACD35698), TbTx5 (P0C5K8) and LmNaTx10 (ACD35698) are classifed as α-toxins based solely on sequence similarity. Cv = *Centruroides vittatus*, Ce = *Centruroides elegans*, Tst = *Tityus stigmurus*, Ts = *Tityus serrulatus*, Pg = *Parabuthus granulatus*, Tb = *Tityus bahaensis*, Lm = *Lychas mucronatus*. GenBank accession numbers are shown in parentheses following the toxin name.

An alignment of the N-terminal sequence obtained from Edman degradation of the CvIV4 peptide with a translation of the nucleotide sequence obtained from *C. vittatus* venom gland cDNA shows that we isolated the gene that encodes CvIV4 (Fig. 4A). Of the initial 40 amino acid residues from the peptide, 35 are identical to the gene translation (five residues at the C-terminal end of the peptide could not be identified using Edman degradation). In addition, the first four cysteines are located in the same positions in both the peptide and translated gene. BLAST (NCBI) searches using both the CvIV4 nucleotide sequence and translated protein identified similar Na^+^ channel toxins from several other scorpion species. An alignment of the translated gene for CvIV4 with toxins from seven other species shows that CvIV4 is most similar to CeII8, a Na^+^ channel toxin isolated from the venom of the Mexican scorpion *Centruroides elegans* (Fig. 5). CvIV4 and CeII8 share 64% of their amino acid residues. The first seven amino acids from both toxins are identical and seven of their eight cysteines (cysteines 1, 2, 3, 4, 5, 7, 8) are located in the same positions. The location of cysteine 6 differs by only one residue position. Interestingly, CeII8 has a primary structure similar to other scorpion α-toxins, but is classified as a beta (β) toxin based on electrophysiological assays [27]. CvIV4 shares over 50% of its amino acid residues with six additional scorpion Na^+^ channel toxins (Fig. 5). The initial seven amino acid residues from these toxins are highly conserved and their eight cysteines are located in similar positions. Ts3 and TsV, isolated from the venom of the South American scorpion *Tityus serrulatus*, are classified as α-toxins based on electrophysiological data. Tst3 and TbTx5, isolated from the South American scorpions *T. stigmurus* and *T. bahiensis* respectively, are classified as α-toxins based solely on sequence similarity to other α-toxins. Pg8, isolated from the African scorpion *Parabuthus granulatus*, and LmNaTx10, isolated from the Asian scorpion *Lychas mucronatus*, are also classified as α-toxins based solely on sequence similarity.

### Effects of CvIV4 on VGSC subtypes

#### Effects of CvIV4 on Na^+^ current

**Figure 6:**
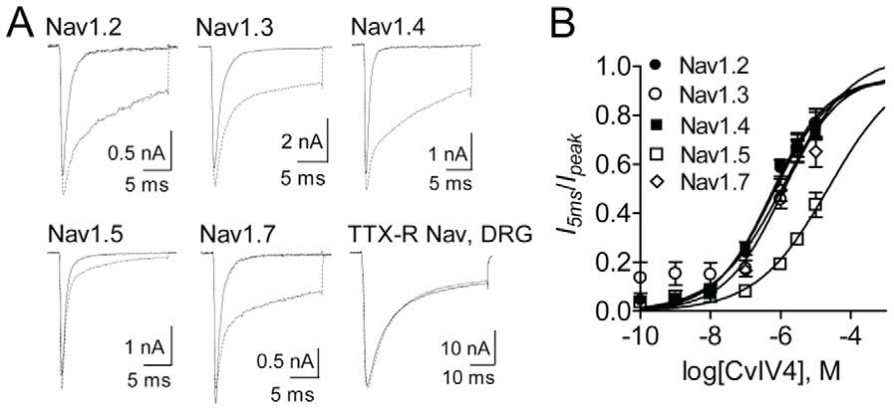
Effects of toxin CvIV4 on voltage-gated sodium channel isoforms. A. CvIV4 (1 µM) slowed the fast inactivation of isoforms Na_v_1.2, Na_v_1.3, Na_v_1.4 and Na_v_1.7 expressed in human embryonic kidney cells (HEK). In contrast, CvIV4 had a minimal effect on Na_v_1.5 (expressed in HEK) and no effect on neuronal TTX-R sodium current isolated from adult rat dorsal root ganglia (DRG) neurons (500 nM TTX was used to block TTX-S sodium current). All sodium current traces were elicited by depolarizing to −10 mV from a holding potential of −100 mV. B. Dose-response curves for CvIV4 slowing the fast inactivation of five sodium channel isoforms (Na_v_1.2–1.7).

CvIV4 induces pain in mammals. Pain sensation is regulated, in part, by three VGSC subtypes (Na_v_1.7, Na_v_1.8, Na_v_1.9) that are expressed in nociceptors. We tested CvIV4 on hNa_v_1.7 expressed in HEK cells and on dissociated rat DRG, which express all three subtypes. Na_v_1.7 is tetrodotoxin sensitive (TTX-S) while Nav1.8 and Nav1.9 are tetrodotoxin resistant (TTX-R). Cells were depolarized to −10 mV from a holding potential of −100 mV and Na^+^ current was measured before and after the application of CvIV4. A comparison of pre- and post-toxin current traces shows that CvIV4 slowed the fast inactivation of Na_v_1.7, prolonging Na^+^ current through the pore (Fig. 6A). However, CvIV4 had no effect on TTX-R current recorded from DRG. As Na_v_1.9 currents disappear very quickly under standard recording conditions in DRG neurons, the majority of the TTX-R current recorded from the DRG neurons reflects the activity of Na_v_1.8 currents. We did not see evidence that CvIV4 enhanced Na_v_1.9 currents in the DRG neurons. Thus, the results identify Na_v_1.7 as the target of CvIV4, demonstrate that Na_v_1.8 is not a target and suggest that CvIV4 does not enhance activity of Na_v_1.9 currents.

Our molecular analyses of *C. vittatus* venom gland mRNA showed that CvIV4 has a primary structure similar to α-toxins produced by other scorpions. Scorpion α-toxins slow fast inactivation in VGSCs and prolong Na^+^ current through the pore. By slowing inactivation and prolonging current in Nav1.7, the results confirm that CvIV4 functions as an α-toxin.

We tested CvIV4 on four additional Na^+^ channel isoforms expressed in HEK cells (rNa_v_1.2, rNa_v_1.3, rNa_v_1.4, hNa_v_1.5). CvIV4 (1 µM) slowed the fast inactivation and prolonged current in Na_v_1.2, Na_v_1.3, and Na_v_1.4, but had minimal affect on Na_v_1.5 (Fig. 6A). The dose-response curves for CvIV4 show that the toxin produced the strongest effects on Na_v_1.2 and Na_v_1.4, moderately strong effects on Na_v_1.3 and Na_v_1.7, and weak effects on Na_v_1.5 (EC_50_ values, in µM: Na_v_1.2, 0.58; Na_v_1.3, 1.31; Na_v_1.4, 0.53; Na_v_1.5, 23.3; Na_v_1.7, 1.34; Fig. 6B). These results show that CvIV4 is not selective for Na_v_1.7.

#### Effects of CvIV4 on Nav1.7

**Figure 7:**
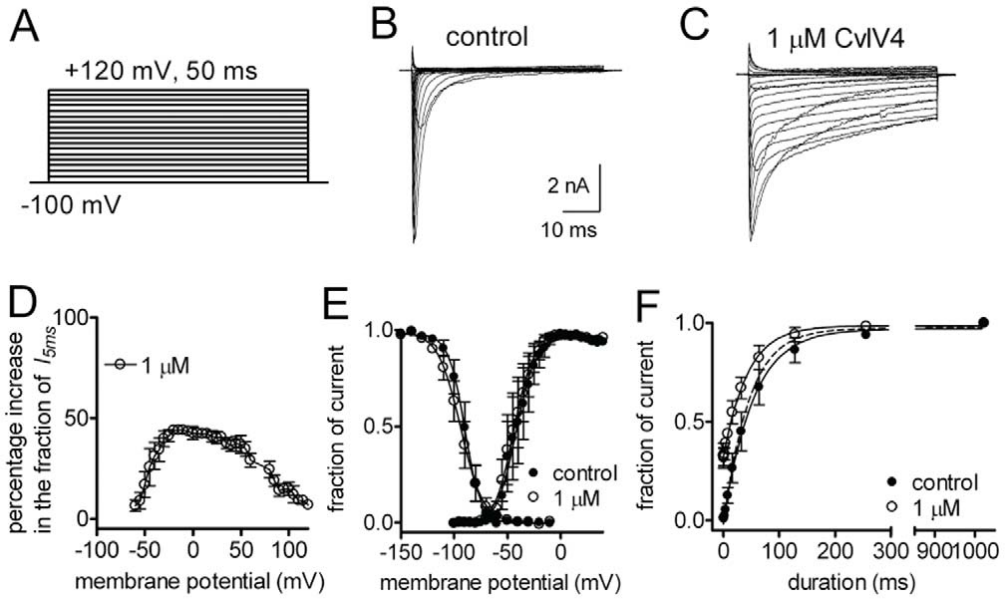
Effects of toxin CvIV4 on activation and inactivation of isoform Na_v_1.7 expressed in Hek293 cells. A. Depolarizing pulses in 5-mV increments were used to elicit current from Na_v_1.7 expressed in HEK cells before (B) and after (C) the application of 1 µM CvIV4. D. Effects of 1 µM toxin on the current-voltage relationship of Na_v_1.7. Currents not inactivated at 5 ms in the presence of 1 µM toxin were plotted as the percentage increase in the fraction of current at 5 ms (*I_5 ms_*). E. Effects of CvIV4 on steady-state activation and inactivation of Na_v_1.7. When data points were fitted with a Boltzmann equation, the V1/2 values were −90.2±1.2 and −94.1±1.6 mV before (filled circles) and after (open circles) toxin treatment, respectively. F. Effect of CvIV4 on rate of recovery from inactivation at −100 mV. Cells held at −100 mV were given a depolarizing prepulse to 0 mV for 20-ms followed by a step back to −100 mV with increasing duration to allow channels to recover from inactivation, followed by a 20-ms test depolarization of 0 mV to activate those channels that had recovered from inactivation. Data points presented as a fraction of the maximum recovered current were fitted with single exponential function to estimate the time constant. The time constants were 58.7±15.4 and 48.2±15.3 ms before (filled circles) and after (open circles) toxin treatment, respectively.

We wanted to know if CvIV4 had additional effects on the voltage-dependent properties of Na_v_1.7. To test this, HEK cells expressing Na_v_1.7 were held at −100 mV and then depolarized for 50 msec, in 5 mV steps, to +120 mV (Fig. 7A). Na^+^ currents were measured before (Fig. 7B) and after (Fig. 7C) the application of CvIV4. A comparison of current traces pre- and post-toxin showed that CvIV4 prolonged the flow of Na^+^ current through the channel, but did not affect the peak amplitude of current. The fraction of peak current remaining at 5 ms was approximately 0.5 (I_5 ms_). A current-voltage (I–V) plot comparing peak currents pre- and post-toxin with the fraction of current remaining at 5 ms (post-toxin) showed differences in the amount of current remaining at different membrane potentials (data not shown). This suggested that there might be a voltage-dependent effect on toxin binding. To address this, we plotted the percentage increase in the fraction of current at 5 ms (post-toxin) vs. membrane potential (Fig. 7D). The results showed that effects of the toxin increased from −60 mV to reach a peak at approximately −30 mV. The effects of the toxin began to decrease at 0 mV, reaching the lowest point around 100 mV. The results demonstrate that the toxin requires depolarizing potentials to slow channel inactivation, however, strong depolarizing potentials decrease the effects of the toxin.

While CvIV4 slowed channel inactivation, it had no effect on the voltage dependence of steady-state inactivation (Fig. 7E). The effect of CvIV4 on the voltage-dependence of activation was minimal, as the membrane potential was shifted by only −4 mV (Fig. 7E).

We also analyzed the effects of CvIV4 on the rate of recovery from inactivation. Cells were held at −100 mV and then given a depolarizing prepulse to 0 mV for 20 msec, followed by a step back to −100 mV with increasing duration to allow channels to recover from inactivation. This was followed by a 20 msec test pulse to 0 mV to activate those channels that had recovered from inactivation. The results demonstrated that 1 µM of toxin decreases the time constant for recovery by approximately 20% (Fig. 7F).

#### Effects of CvIV4 on Na_v_1.2, 1.3, 1.4, 1.5

**Figure 8:**
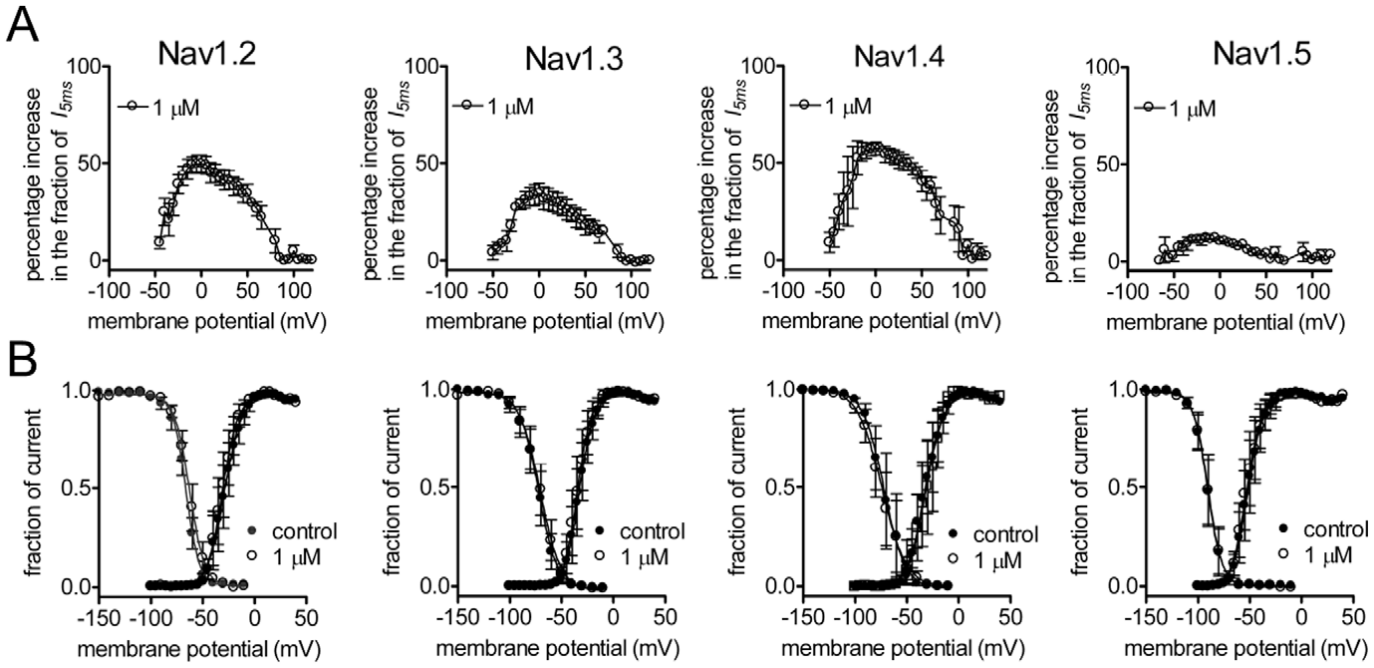
Effects of CvIV4 on activation and inactivation of Na_v_1.2, Na_v_1.3, Na_v_1.4 and Na_v_1.5. 50-ms depolarizing pulses were used to elicit Na^+^ current from channel isoforms expressed in HEK cells before and after the application of 1 µM CvIV4. Cells were held at −100 mV. Depolarizing potentials ranged from −100 to +120 mV in 5-mV increments. A. Currents not inactivated at 5 ms in the presence of 1 µM toxin were plotted as the percentage increase in the fraction of current at 5 ms (*I_5 ms_*). B. Effects of CvIV4 on steady-state activation and inactivation of Na^+^ channel isoforms.

CvIV4 also prolonged Na^+^ current through four additional VGSC isoforms (Na_v_1.2, Na_v_1.3, Na_v_1.4, Na_v_1.5). Plots of the percentage increase in current at 5 ms (post-toxin) against membrane potential showed that the effects of CvIV4 are voltage-dependent (Fig. 8A). The results also showed that CvIV4 differed in its effects depending on the channel subtype. Na_v_1.3, Na_v_1.2 and Na_v_1.4 showed increases in the fraction of current present at 5 ms (I_5 ms_) that peaked at 40%, 50%, and 60%, respectively. The peak amplitude occurred at approximately 0 mV for all three subtypes and strong depolarizing potentials (+50 to +100 mV) decreased the effects of the toxin. In contrast, CvIV4 had minimal effects on Na_v_1.5, with the fraction of current at 5 ms reaching a peak of only 10% around −20 mV.

We tested the effects of CvIV4 on the voltage-dependence of activation and inactivation for Na_v_1.2, Na_v_1.3, Na_v_1.4 and Na_v_1.5. A comparison of the fraction of current recorded over a range of membrane potentials (pre- and post-toxin) for all four subtypes shows that CvIV4 does not shift the voltage-dependence of either activation or inactivation for any of the subtypes (Fig. 8B).

### Comparison of CvIV4 with scorpion toxins that target Na_v_1.7

**Figure 9:**
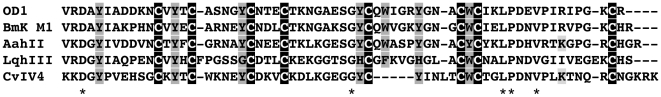
Comparison of CvIV4 with Old World α-toxins that modulate Na_v_1.7. CvIV4 is aligned with ODI, BmK MI, AahII and LqhIII, Old World α-toxins that slow the fast inactivation of Na_v_1.7. The biological activity of CvIV4 is similar to these peptides, but its primary structure is not. Gaps were introduced to align cysteine residues (white font with black background). Hydrophobic residues (dark shaded background) that are critical for α-toxin structure and function are conserved in CvIV4 at positions 5, 14, 22, 36, 43 and 49 [28]. CvIV4 shares a lysine (light shaded background) with AahII (position 60) that is critical for the biological activity of AahII [29]. Additional amino acids that are identical among toxins are marked with an asterisk (*). GenBank accession numbers: CvIV4 (JF938594), ODI (P84646), BmK MI (P45697), AahII (P01484), LqhII (P59355). MAFFT version 6 used for sequence alignment (http://mafft.cbrc.jp/alignment/software/).

CvIV4 has a primary structure similar to seven other scorpion toxins (Fig. 5). However, of these seven toxins, only CeII8 has been tested on Na_v_1.7. The remaining six toxins have not been tested on Na_v_1.7. We wanted to compare the sequence for CvIV4 with other scorpion toxins that modulate Na_v_1.7. A literature search revealed four additional toxins isolated from Old World scorpions [ODI, *Odonthobuthus doriae*; AahII, *Androctonus australis* Hector; LqhIII, *Leiurus quinquestriatus hebraeus*; BmK MI (BmK I), *Buthus martensii* Karsch] that target Na_v_1.7 [19], [20]. Interestingly, all of these peptides function as α-toxins, slowing the fast inactivation and prolonging Na^+^ current through Na_v_1.7. While the biological activity of these toxins is similar to CvIV4, an alignment of CvIV4 with ODI, AahII, LqhIII and BmK MI (BmK I) showed that the primary structure of CvIV4 is not similar to these Old World α-toxins (Fig. 9). However, the number and position of cysteines is conserved. In addition, there are six hydrophobic amino acids that are conserved between CvIV4 and these Old World toxins that correspond to positions important for the structure and function of α-toxins [28]. CvIV4 also shares a lysine with AahII (position 60, Fig. 9) that is critical for the biological activity of AahII [29].

### Localization of amino acids critical for CvIV4 activity

**Figure 10:**
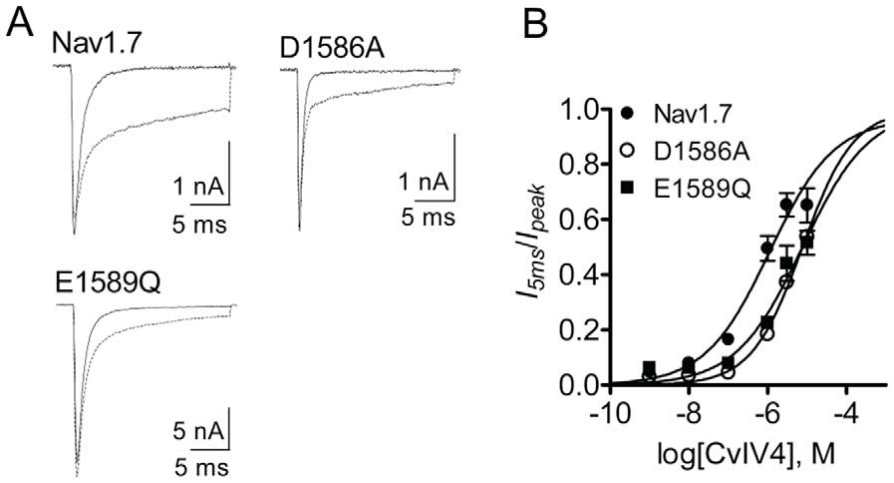
Effects of CvIV4 on mutant Na_v_1.7 channels expressed in HEK293 cells. A. Substitution of negatively charged amino acids in the Domain IV S3–S4 loop with neutral residues (D1586A, E1589Q) reduces the effects of 1 µM CvIV4 on Na_v_1.7. B. Dose-response curves for CvIV4 on wildtype and mutant Na_v_1.7 channels.

Scorpion α-toxins bind to VGSCs at site 3 (Domain IV S3–S4 loop) [28], [30]. To localize the residues in the DIV S3–S4 loop that are critical for CvIV4 activity, we made two mutants of hNa_v_1.7 channels and expressed them in HEK cells. The acidic residue Asp (D, position 1586 in hNa_v_1.7) is important for α-toxin activity. We substituted the negatively charged Asp with a neutral Ala (D1586A) and decreased the effect of CvIV4 on hNa_v_1.7 (Fig. 10A). The acidic residue Glu (E, position 1589 in hNa_v_1.7) is also critical for α-toxin activity. Glu occurs at this same position in Na_v_1.3 and Na_v_1.2, also targets of CvIV4. However, in Na_v_1.4 (target of CvIV4) a Gln (Q) occurs at this position. We substituted the Glu in hNa_v_1.7 with Gln (E1589Q) and reduced the effects of CvIV4 (Fig. 10A). The results suggest that both Asp1586 and Glu1589 are important for the effects of CvIV4 on Na_v_1.7. However, while both substitutions reduced the effects of CvIV4 on Nav1.7, neither abolished the effects of this toxin, which suggests other residues in DIV S3–S4, or other channel regions, are necessary for the activity of CvIV4.

## Discussion

### Differences in the pain-inducing capability of venom

Anecdotal reports suggest that the stings of buthid scorpions are intensely painful, more so than the stings of other scorpions. However, no studies have used a mouse model to quantify and compare the pain induced by buthid and non-buthid venoms [16], [21], [31]. We found that the venom of one species of scorpion from the family Vaejovidae (*V. spinigerus*), whose sting is reported to be moderately painful in humans, caused significantly more paw licking in mice than did an injection of water. However, the venom of two buthid scorpions (*C. vittatus* and *C. exilicauda*) induced significantly more paw licking in mice than *V. spinigerus* venom. These results demonstrate that vaejovid venom is painful to mammals, however, the results agree with anecdotal reports that buthid venom is considerably more painful. The results also show that the sensation of pain produced by buthid stings is conserved across mammals. We also observed differences between the two species of *Centruroides* in their pain-inducing capability. Such variability in painfulness may reflect different ecological and/or evolutionary histories of these scorpions with their predators [32], [33].

### Pain-inducing components in C. vittatus venom (CvIV4)

We screened fractions of *C. vittatus* venom for pain-inducing capability and identified four fractions that produced paw licking in mice. One of those fractions (P4) caused a response in mice similar to the response induced by the whole venom. Fraction P4 contained four peptides, three of which produced paw licking. Of those three, P4-4 (CvIV4) produced more than twice as much paw licking as the other two peptides. While CvIV4 generated more paw licking than the other peptides, its effects were not immediate, but commenced between 30 and 60 seconds following an injection. Moreover, paw licking was sporadic during the first minute following the injection and did not reach a peak until the third and fourth minutes of the test. In contrast, injections of either whole venom or fraction P4 produced an immediate response. Humans report that *Centruroides'* stings produce immediate, intense pain. This suggests that other components in *C. vittatus* venom may be necessary for producing an immediate response. While the other three peptides in fraction P4 (P4-1, P4-2, P4-3) did not generate as much paw licking as CvIV4, they may be responsible for initiating an immediate pain response. Fractions P1, P2 and P5 produced low to moderate amounts of paw licking, while fraction P3 failed to produce a response. It is possible that one or more of these fractions, either individually or in combination, are critical for initiating an immediate pain response in mammals.

### Structural characteristics of CvIV4

The molecular mass and the initial 40 amino acids of the N-terminal sequence for CvIV4 are characteristic of scorpion toxins that bind Na^+^ channels [6], [34]. Scorpion toxins that bind voltage-gated Na^+^ channels are polypeptides composed of a single strand of amino acids. The peptides range from 58 to 76 amino acids in length (6500–8500 amu) and they contain eight cysteines that form four disulfide bonds. The conserved structural scaffold of these peptides consists of one α-helix and two or three strands of β-sheet, typically arranged in the order βαββ. We isolated cDNA clones from *C. vittatus* venom gland mRNA and identified a clone whose translated nucleotide sequence matched the N-terminal amino acid sequence of CvIV4. A comparison of the translated cDNA with sequences from other scorpion toxins confirmed that CvIV4 is a Na^+^ channel toxin.

Na^+^ channel toxins can be divided into two groups (alpha and beta) based on their functional effects [6], [7]. Beta (β) toxins shift the voltage-dependence of activation to more negative potentials, making the channel more likely to open at membrane potentials where activation would normally not occur. Alpha (α) toxins inhibit the fast inactivation mechanism, prolonging Na^+^ current through the channel. While α- and β-toxins are structurally conserved, they are not identical. A BLAST search (NCBI) revealed that the primary structure of CvIV4 is most similar to the structure of venom peptides classified as α-toxins [6], [28], [35]. CvIV4 contains eight cysteine residues whose pattern is highly similar to other scorpion α-toxins. In addition, the composition and location of amino acid residues corresponding to the α-helix and β-sheets conserved among α-toxins are also observed in CvIV4. The primary structure of CvIV4 is most similar to CeII8, a toxin recently isolated from the venom of *Centruroides elegans* [27]. CvIV4 and CeII8 share 64% of their amino acids, however, CeII8 is classified as a β-toxin based on its functional effects.

### Functional characteristics of CvIV4

Noxious stimuli activate nociceptors located in the peripheral sensory pathway. Nociceptors express three different VGSC subtypes (Na_v_1.7, Na_v_1.8, Na_v_1.9) that play a role in regulating pain perception. We tested CvIV4 on hNa_v_1.7 expressed in HEK cells and on whole cell Na^+^ currents recorded from rat DRG. We found that the toxin slowed the fast inactivation of hNa_v_1.7, however, CvIV4 had no effect on TTX-R currents recorded from DRG. Na_v_1.7 is TTX-S, while both Na_v_1.8 and Na_v_1.9 are TTX-R. Thus our findings demonstrate that CvIV4 targets Na_v_1.7 and not Na_v_1.8. Although the pharmacology of Na_v_1.9 currents are harder to study due to pronounced current run-down, our data also suggest that CvIV4 does not enhance Na_v_1.9 currents. This suggests that CvIV4 induces pain in mammals by slowing the fast inactivation of Na_v_1.7.

By slowing the fast inactivation of Na_v_1.7, CvIV4 induced a persistent Na^+^ current, but did not change the peak amplitude of the current. The persistent Na^+^ current could be abolished by strong depolarizing membrane potentials, which suggests that CvIV4 dissociates from its target at highly positive membrane potentials. CvIV4 had no effect on the voltage-dependence of inactivation and shifted the voltage-dependence of activation to the left by only 4 mV. All of these effects are characteristic of the activity reported for scorpion α-toxins [7], [17], [19], [20], [28], [30], [36] confirming that CvIV4 functions as an α-toxin. Moreover, these findings provide a mechanism to explain how CvIV4 produces the sensation of pain in mammals. By prolonging Na^+^ current in Na_v_1.7, CvIV4 would cause nociceptors to become hyper-excitable, thus producing the sensation of pain.

Purified samples of CvIV4 induced paw licking in mice, however, the response was delayed as compared to whole venom or fraction P4. It is possible that one of the other fractions or peptides isolated from *C. vittatus* venom is necessary for initiating immediate pain sensation. A recent study of *Centruroides elegans* venom components showed that CeII8 binds Na_v_1.7 expressed in *Xenopus* oocytes [27]. In contrast to CvIV4, CeII8 acts as a β-toxin and shifts the voltage-dependence of activation to more negative potentials, making Na^+^ channels more likely to open. Because β-toxins make Na^+^ channels more likely to open at subthreshold membrane potentials, it is plausible that β-toxins are responsible for inducing the immediate sensation of pain associated with scorpion stings. However, the study of *Centruroides elegans* venom did not directly test whether CeII8 produces paw licking in mammals.

Results from the electrophysiology data showed that micro-molar concentrations of CvIV4 were required to slow the inactivation of Na_v_1.7. However, we do not know the concentration of CvIV4 delivered in a single sting. Nor do we know whether or how CvIV4 interacts with other toxins in the venom to produce the sensation of pain in rodents. Interestingly, a recent study in *Xenopus* oocytes indicated that sodium channel β subunits can modulate the affinity of Na_v_1.8 channels for conotoxins [37]. Other studies have reported that β subunits do not alter the interaction of sodium channels with neurotoxins in mammalian cells [38]. It is possible that specific β1–4 subunit combinations might alter the affinity of specific sodium channel isoforms for CvIV4. Unfortunately, it is not known which β subunits associate with Na_v_1.7 in DRG neurons.

CvIV4 also slowed the fast inactivation and produced a persistent Na^+^ current in rNa_v_1.2, rNa_v_1.3 and rNa_v_1.4. However, CvIV4 had a minimal affect on hNa_v_1.5. These findings show that CvIV4 is not selective for Na_v_1.7, but instead binds several VGSC isoforms. This is in contrast to CeII8, which is selective for Na_v_1.7 [27]. Given that CvIV4 and CeII8 are similar in structure but differ in function and isoform selectivity, a comparison of these two toxins should improve our understanding of the structural characteristics that determine New World scorpion toxin selectivity and function.

Four Old World α-toxins, ODI, isolated from the Iranian scorpion *Odonthobuthus doriae*, AahII, from the African scorpion *Androctonus australis* Hector, LqhIII, from the Middle Eastern scorpion *Leiurus quinquestriatus hebraeus* and BMK MI (BmK I), from the Asian scorpion *Buthus martensii* Karsch, all slow the inactivation of Na_v_1.7 [16], [17], [19], [20], [31]. Anecdotal reports suggest that all four of these scorpion species produce painful stings. However, of the four toxins, only BmK MI (BmK I) has been tested for its pain-inducing capability in mammals. While the biological activity of CvIV4 is similar to these four toxins, the primary structure of CvIV4 is not similar. However, eight cysteines and six hydrophobic amino acids that are critical for the structure and function of α-toxins are conserved between CvIV4 and these four toxins [28]. Moreover, CvIV4 shares a lysine residue with AahII that is important for the activity of AahII [29]. These results raise intriguing questions about the evolution of pain-inducing toxins in buthid scorpions. Old and New World scorpions diverged from a common ancestor approximately 150 million years ago [39]. It is possible that pain-inducing toxins evolved before the split between Old and New World scorpions, and were then conserved. Alternatively, pain-inducing toxins may have evolved separately in both groups since their divergence. Identification and comparison of additional pain-inducing toxins from both Old and New World scorpions would provide insight into the evolution of these toxins in Buthidae scorpions.

For scorpions like *C. vittatus* that are small and have slender pincers, venom that induces pain may provide the opportunity to escape from potential predators. In a series of staged feeding trials, wild-caught grasshopper mice (*Onychomys arenicola*, *O. torridus*), voracious predators of scorpions, were fed a variety of their local prey items, including *Centruroides* spp., *Vaejovis* spp. and field crickets [32]. Both *O. arenicola* and *O. torridus* dropped *C. vittatus* and *C. exilicauda* (respectively) significantly more often than they dropped the *Vaejovis* species or the crickets. This suggests that CvIV4 may function as a defense against predators of *C. vittatus*.
